# Acupoint catgut embedding for chronic low back pain: A protocol for systematic review and meta-analysis

**DOI:** 10.1097/MD.0000000000032409

**Published:** 2022-12-30

**Authors:** Guofu Zhong, Xiuju Yin, Jingyi Li, Xiaohui Li, Qiang Zhang

**Affiliations:** a Shenzhen Traditional Chinese Medicine Hospital, Shenzhen, Guangdong, China; b The Second Affiliated Hospital, Guangzhou University of Chinese Medicine, Guangzhou, China; c Shunde Hospital of Guangzhou University of Chinese Medicine, Guangzhou University of Chinese Medicine, Guangzhou, Guangdong, China; d Shenzhen Hospital of Beijing University of Chinese Medicine, Guangdong, China.

**Keywords:** acupoint catgut embedding, chronic low back pain, protocol, systematic review

## Abstract

The treatment of acupoint catgut embedding (ACE) effective and safe for patients with chronic low back pain (CLBP) is not yet known. This systematic review will objectively and systematically evaluate the efficacy and safety of ACE in CLBP according to the existing evidence.

**Methods:**

The protocol of this systematic review and meta-analyses has been registered in PROSPERO with the registration number CRD42019142256. The following electronic databases from inception to November 29, 2022 will be searched: PubMed, the Cochrane Central Register of Controlled Trials (CENTRAL), EMBASE, Web of Science, China National Knowledge Infrastructure (CNKI), Wan Fang Data and Chinese Science Journal Database. Randomised controlled clinical(RCTs) using ACE to treat CLBP will be included. Outcomes will include pain intensity, instrument with assessment function and disability, quality-of-life, and costs. Adverse events will be reported for safety assessment. By screening the titles, abstracts, and full texts, two independent reviewers will select studies, extract data, and assess study quality. Data synthesis, sensitivity analysis, subgroup analysis and risk of bias assessment will be conducted using RevmanV.5.3 software. The Grading of Recommendations Assessment, Development and Evaluation system will be used to assess the quality of

the evidence.

**Results:**

The efficacy and safety of ACE in the treatment of CLBP has not yet been determined.

**Conclusion:**

This systematic review will objectively and systematically evaluate the efficacy and safety of ACE in CLBP according to the existing evidence, which can give high level clinical recommendations to improve patient care and clinical outcomes.

## 1. Introduction

Chronic low back pain (CLBP) is one of the highest ranked condition of years lived with disability, according to the recent global burden of disease study.^[1]^ As a persistent condition, CLPB is commonly defined as the pain occurring in the area of the low back and lasting for at least 3 months.^[2]^ The lifetime prevalence of low back pain (LBP) in general population ranges between 51.0% and 84.0%, in which the prevalence of CLBP is between 5.9 and 18.1%. Being the leading cause of disability, CLBP not only brings enormous inconvenience to the daily life and social communication of patients, but also imposes a heavy burden and huge workload on families and society.^[3,4]^ Treatment for LBP is targeted and specific according to the patient’s reality, aiming mainly at preventing or reducing disability, and returning to physical and social activities as soon as possible.^[5]^ For acute LBP, appropriate bed rest, keep physically active, and symptomatic treatment are recommended. CLBP, on the other hand, is more complex in treatment.^[6]^ The conventional treatment of CLBP includes both pharmacological and non-pharmacological treatments, with pharmacological treatment being the primary choice of clinicians.^[7]^ However, non-pharmacological interventions currently are emphasized over pharmacological therapies due to the inevitable side effects with the long-term use of some medications such as nonsteroidal anti-inflammatory drugs and opioid analgesics.^[8]^ Non-pharmacological therapy, such as psychological therapy, physical therapy, and cognitive functional therapy plays a more and more indispensable role on improving functions, relieving pain, and minimizing disability for patients with CLBP.^[6,9]^ However, owing to the variety of lifestyle and economic condition, it is difficult for patients to follow the treatment protocols of exercise and rehabilitation therapies.^[10]^ As the 2016 CDC Guidelines for Prescribing Opioids for Chronic Pain and the 2017 American College of Physicians clinical practice guidelines recommended, non-pharmacologic interventions and acupuncture can be a first-line management for patients with CLBP.^[11]^ Given the increasing burden of non-pharmacological therapy and the side effects of pharmacological therapy, patients with CLBP increasingly turn to complementary and alternative therapies for supporting, especially acupuncture, due to less side effect and beneficial in the long run.^[12]^

Acupoint catgut embedding (ACE), an alternative treatment evolving from traditional acupuncture, is characterized by implanting a certain section of absorbable catgut suture in acupoints with needles.^[13]^ The catgut, a type of heterogeneous protein, will be gradually softened, decomposed, dissolved, and finally absorbed by subcutaneous tissue and muscle around the acupoints.^[14]^ At present, the analgesic mechanism of ACE is remaining unclear. With a continue absorption and gentle stimulation lasting for several days, ACE may be a desirable remedy for fewer visits to the doctors, and reducing cost and time of treatment compared with traditional acupuncture.^[13]^ According to traditional Chinese medicine, the pathogenesis of pain is the pathogenic qi invading into collaterals and causing the obstruction of the meridians and collaterals. To consolidate and improve the curative effect, acupuncturist would leave the needle in the acupoint for a certain time to enhance and prolong the acupuncture effect. In the accordance with this theory, ACE come into being and developed, replacing traditional needles with catgut, in order to entering the inside to cure diseases. Studies have demonstrated that ACE mainly achieves the effect of “channeling meridians, regulating qi and blood” and “regulating qi and blood” by coordinating and balancing “zang-fu” organs, dredging blood stasis and meridians, and harmonizing the mode of action of Yin and Yang, qi, and blood.^[15]^ Experimental researchers found that ACE could relieve pain and reduce anxiety and depression to achieve physical and mental regulation through the activation of the central neurotransmitter, dopaminergic nervous system, and hypothalamic-pituitary-adrenal axis.^[16]^

ACE, as an alternative therapy, has attracted extensive interest for the advantage minimal invasive, long-lasting therapeutic effect with few sides effect, and has been wildly applied to the treatment of CLBP and other conditions such as musculoskeletal diseases, obesity, premenopausal syndrome, and depressive condition in China, Korea, and other areas.^[17,18]^ Plenty of studies have proven that being an effective supplement, ACE is advantages in alleviating the symptoms of patients with CLBP in a continuous and safe approach, thus it improves quality of life of patients.^[19]^ However, to our knowledge, no systematic review or meta-analysis has been supporting the evidence regarding the efficacy and safety of ACE in the treatment of CLBP. Therefore, we intend to conduct a systematic review evaluating the efficacy and safety of ACE for CLBP based on available evidences.

## 2. Methods

### 2.1. Study registration

This systematic review protocol had been registered on PROSPERO (registration number: CRD 42019142256). The protocol was developed and reported following the Preferred Reporting Items for Systematic Review and Meta-Analysis Protocols.

### 2.2. Criteria for including studies in the review

#### 2.2.1. Type of study.

Randomised controlled trials (RCTs) regarding ACE for CLBP published in English and Chinese will be included without published status.

#### 2.2.2. Type of participants.

Participants aged from 18 to 65 years with a diagnosis of CLBP^[20]^ will be included in the review regardless of gender, race, or economic status.

#### 2.2.3. Type of interventions and comparisons.

ACE used as the sole treatment or in combination with other adjunct treatments for CLBP will be included, such as ACE compared with no treatment, ACE compared with pharmacological therapies (e.g., nonsteroidal anti-inflammatory drugs) or ACE combined with active treatment (e.g., psychotherapy or physical therapy, surgery) compared with the active treatment alone (the same as the ACE group).

#### 2.2.4. Types of outcome measures.

##### 2.2.4.1. Primary outcomes

The pain intensity of chronic low back pain which measured in visual analogy scale, the digital rating scale, the speech rating scale, or the revised facial pain scale will be collected and recorded as the primary outcome.

##### 2.2.4.2. Secondary outcomes

- Functional disability assessment by the Quebec Back Pain Disability or the Roland-Morris Disability Questionnaire;
- Quality of life measurement;
- Costs;
- Adverse events.

### 2.3. Search methods for identification of studies

#### 2.3.1. Electronic searches.

The following electronic databases from their inception to November 29, 2022 will be searched: PubMed, the Cochrane Central Register of Controlled Trials, EMBASE, Web of Science, China National Knowledge Infrastructure, Wan Fang Data and Chinese Science Journal Database. No restrictions will be placed on publication date. The search strategies for all databases are shown in Table 1.

**Table 1:** Search strategy for each database.

Database	Search items
PubMed	1 Low back pain OR backache
	2 acupoint catgut embedding
	1 AND 2
CENTRAL	1 Low back pain OR backache
	2 acupoint catgut embedding
	1 AND 2
EMBASE	1 Low back pain OR backache
	2 acupoint catgut embedding
	1 AND 2
Web of Science	1 Low back pain OR backache
	2 acupoint catgut embedding
	1 AND 2
CNKI	1 腰痛 OR下腰痛
	2 穴位埋线OR埋线
	1 AND 2
Wan Fang	1 腰痛 OR下腰痛
	2 穴位埋线OR埋线
	1 AND 2
Chinese Science Journal Database	1 腰痛 OR下腰痛
	2 穴位埋线OR埋线
	1 AND 2

CENTRAL = Central Register of Controlled Trials, CNKI = China National Knowledge Infrastructure.

#### 2.3.2. Searching other resources.

We will also screen the reference lists of identified relevant RCTs, previous systematic reviews for additional relevant trials. The US National Institutes of Health Ongoing Trials Register (https://clinicaltrials.gov/), the Chinese Clinical Trial Registry (ChiCTR), the WHO International Clinical Trials Registry Platform (https://www.who.int/clinical-trials-registry-platform) and Google Scholar will also be retrieved for any ongoing, planned, or unpublished relevant RCTs. Meanwhile, if necessary, we will contact experts in the relevant fields for the unreported of ACE for CLBP.

### 2.4. Data collection and analysis

#### 2.4.1. Selection of studies.

**Figure 1.**
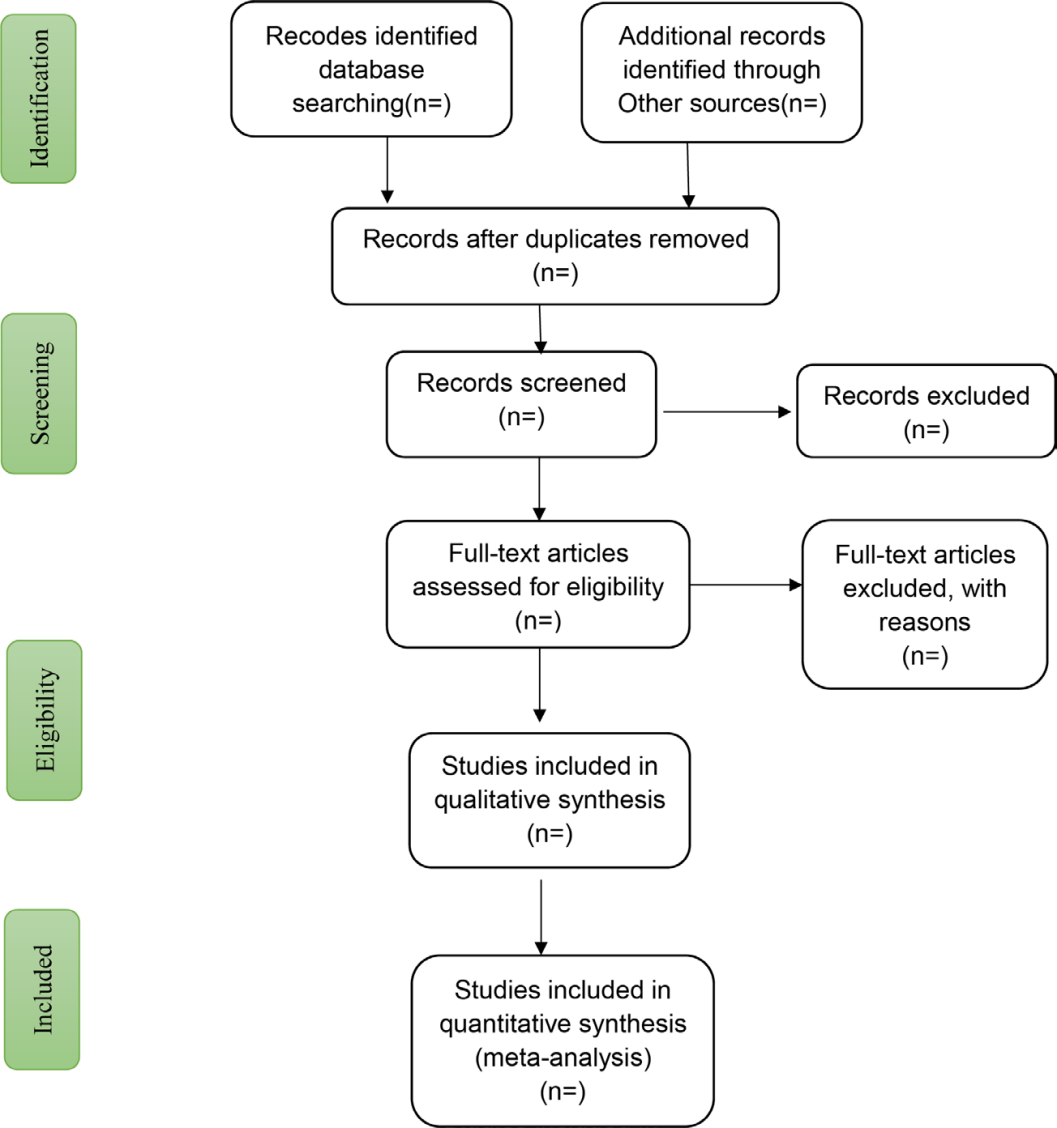
Flow diagram of study selection process.

Two independent reviewers will first search titles and abstracts of potential studies from the selected records and import all relevant studies into a database created by Endnote X8 with double checking. Two reviewers will further evaluate the potential studies by reading full text based on the inclusion criteria. All excluded studies will be listed in a table with eliminate reasons. The screening results will be cross-checked between the 2 independent reviewers. Any disagreement will be resolved in a discussion or arbitrated by an experienced reviewer. The selection procedure will be demonstrated in a PRISMA flow diagram (Fig. 1).

#### 2.4.2. Data extraction and management.

All data extraction of eligible studies will be undertaken by 2 independent reviewers and preserved in a standard data extraction form designed and worked out by all researchers. The data extraction form will include the following items: general information (author, working location, publication date, journal, etc), participants characteristics (age, sex, sample size, disease course), design of interventions (type, duration, follow-up, compliance), outcomes, characteristics of research methodology (randomization, allocation concealment, incomplete data, blinding, selective report), adverse events, and conflicts of interest. All extraction will be checked repeatedly by the 2 extractors. Any discrepancy occurred during the process will be discussed between the 2 extractors or adjudged by a third reviewer. When the data are not available or ambiguous, we will email or telephone to the corresponding authors of the RCTs for detail information.

### 2.5. Assessment of risk of bias in included studies

The risk of bias of each eligible trail in the review will be appraised and cross checked by 2 reviewers independently in the accordance of the Cochrane Collaboration’s ‘Risk of Bias’s Tool by the following 6 domains: sequence generation, allocation concealment, blinding of the outcome assessment, incomplete data assessment, selective outcome reporting and other sources of bias. The assessment will be categorized into 3 levels: low risk of bias, unclear risk of bias and high risk of bias. Any disagreement in evaluation will be addressed by discussion or consulting another experienced reviewer. Items that are not clear in the trials will be asked for details by contacting the correspondent authors.

### 2.6. Measures of treatment effect

RevMan V.5.3 will be used to compute the outcome data. For dichotomous data, we will express the efficacy data as risk ratio with 95% CIs. For continuous data, on the other hand, mean difference with 95% CIs will be used to present the estimate of the effect instead.

### 2.7. Dealing with missing data

Researchers will attempt to contact the corresponding authors of original trials to obtain missing data. Failure to obtain the data, we will conduct an intention-to-treat analysis, and if propriety, a sensitivity analysis will be performed to explore the potential impact of missing data.

### 2.8. Assessment of heterogeneity

We will use *χ*^2^ test and *I*^2^ statistic in the forest plots conducted by the RevManV.5.3 to assess statistical heterogeneity of the original studies from different sources, and the *χ*2 test with a *P* value less than 0.10 or *I*^2^ > 50% will indicate statistical heterogeneity. A subgroup analysis will be performed to detect the possible causes in the case of significant heterogeneity is observed.

### 2.9. Unit of analysis issues

We will include studies with multiple intervention groups. Comparison will be made between each intervention group and the single control group.

### 2.10. Assessment of reporting biases

If the total amount of included studies is more than 10, then we will judge the publication bias by constructing funnel plots. On the contrary, the evaluation of reporting biases will not be carried out due to the insufficient quantity of included literature (less than 10 trials).

### 2.11. Data synthesis

The data synthesis will be carried out using the Revman V.5.3 software. Meta-analysis will be conducted if the data is suitable. For dichotomous data, we will use risk ratio with 95% CIs, and for continuous data, mean difference with 95% CIs will be adopted. If the clinical outcome measured in different assessment tools, we will use the standardized mean difference with 95%CIs, or weighted mean difference instead. If little or no statistical heterogeneity is detected with *I*^2^ < 50%, the fixed-effects model will be conducted for the pooled data. While there is significant heterogeneity across studies with *I*^2^ ≥ 50%, a random-effect model will be used for data synthesis, and meta-analysis will not be performed. In this case, we will further conduct subgroup analysis or sensitivity analysis to explore potential explanations for heterogeneity.

### 2.12. Subgroup analysis

Subgroup analysis will be conducted to explore the statistical heterogeneity under the available data from clinical and methodological source. Clinical factors such as different control interventions, duration of treatment, outcomes, and methodological factors like the object of the blinding, the means of allocation concealment and the measurement of the endpoint will be investigated.

### 2.13. Sensitivity analysis

We will perform sensitivity analysis by excluding some studies classified as “high risk” of bias to identify the robustness and reliability of the review findings. If there is no obvious change in the results after exclusion, the sensitivity is low and the results are robust and credible. If the results are significant change, we will make a conclusion of high sensitivity and the robustness of our findings is low.

### 2.14. Summary of evidence

The quality assessment of evidence will be conducted using the Grading of Recommendations Assessment, Development and Evaluation (GRADE) approach.^[21]^ The total quality of the evidence of each outcome will be divided into high, moderate, low, and very low quality 4 levels.

### 2.15. Ethics and dissemination

This systematic review will not concern about information of individual patient, and as a result does not request to obtain approval from ethical committee. The findings of this review may be published in a peer-reviewed journal or presented at a relevant conference.

### 2.16. Amendments

The information will be described in the final report, and if the protocol is modified.

## 3. Discussion

The benefits of ACE intervention on CLBP are controversial. The highly heterogeneous results of RCTs and non-randomized interventional studies conducted in recent years have challenged the use of ACE in these patients.^[11]^ The current study has several strengths. Firstly, the review will synthesize a board range of evidence on patients with CLBP from all the available evidence, which can objectively and systematically evaluate the efficacy and safety of ACE for people with a CLBP and thus offer objective evidence for clinicians, patients, and policy-makers on the use of ACE therapy in those patients. Secondly, To the best of our knowledge, this will be the first ever systematic review analyzing ACE intervention in the treatment of CLBP.

However, there are some limitations of this study that need to be discussed. Firstly, simple size, high heterogeneity, high risk of bias to provide high-quality evidence. In addition, we may not have ample evidence to prove efficacy at CLBP caused by different sources. Nonetheless, the outcomes of this review will provide the latest evidence for the efficacy of ACE in treating CLBP, which may benefit clinicians, patients, and policy-makers.

## Author contributions

**Conceptualization:** Guofu Zhong.

**Data curation:** Guofu Zhong, Jingyi Li, Xiaohui Li.

**Funding acquisition:** Xiuju Yin.

**Investigation:** Xiuju Yin.

**Methodology:** Guofu Zhong, Xiuju Yin, Jingyi Li, Xiaohui Li, Qiang Zhang.

**Resources:** Xiaohui Li.

**Software:** Xiaohui Li, Qiang Zhang.

**Visualization:** Guofu Zhong, Qiang Zhang.

**Writing – original draft:** Guofu Zhong, Xiuju Yin, Jingyi Li, Qiang Zhang.

**Writing – review & editing:** Guofu Zhong, Qiang Zhang.
